# Molecular Investigations of a Locally Acquired Case of Melioidosis in Southern AZ, USA

**DOI:** 10.1371/journal.pntd.0001347

**Published:** 2011-10-18

**Authors:** David M. Engelthaler, Jolene Bowers, James A. Schupp, Talima Pearson, Jennifer Ginther, Heidie M. Hornstra, Julia Dale, Tasha Stewart, Rebecca Sunenshine, Victor Waddell, Craig Levy, John Gillece, Lance B. Price, Tania Contente, Stephen M. Beckstrom-Sternberg, David D. Blaney, David M. Wagner, Mark Mayo, Bart J. Currie, Paul Keim, Apichai Tuanyok

**Affiliations:** 1 Translational Genomics Research Institute, Flagstaff, Arizona, United States of America; 2 Northern Arizona University, Flagstaff, Arizona, United States of America; 3 Arizona Department of Health Services, Phoenix, Arizona, United States of America; 4 Centers for Disease Control and Prevention, Atlanta, Georgia, United States of America; 5 Menzies School of Health Research, Darwin, Northern Territory, Australia; SBRI, United States of America

## Abstract

Melioidosis is caused by *Burkholderia pseudomallei*, a Gram-negative bacillus, primarily found in soils in Southeast Asia and northern Australia. A recent case of melioidosis in non-endemic Arizona was determined to be the result of locally acquired infection, as the patient had no travel history to endemic regions and no previous history of disease. Diagnosis of the case was confirmed through multiple microbiologic and molecular techniques. To enhance the epidemiological analysis, we conducted several molecular genotyping procedures, including multi-locus sequence typing, SNP-profiling, and whole genome sequence typing. Each technique has different molecular epidemiologic advantages, all of which provided evidence that the infecting strain was most similar to those found in Southeast Asia, possibly originating in, or around, Malaysia. Advancements in new typing technologies provide genotyping resolution not previously available to public health investigators, allowing for more accurate source identification.

## Author Summary

Melioidosis is a bacterial disease caused by percutaneous inoculation, aspiration or ingestion of the soil bacteria *Burkholderia pseudomallei*. Melioidosis is primarily found in Southeast Asia and Northern Australia, and, to a lesser degree, nearby regions. A recent case of melioidosis in Southwestern United States (Southern Arizona) prompted a detailed epidemiological and molecular investigation to discover the source of infection. The authors describe the use of multiple genomic analysis tools to aid in this investigation. The results of these analyses uniformly identified Southeast Asia as the source of the strain that infected the patient, however the epidemiological investigation had determined the patient had no international travel or known exposures to Southeast Asian products. New cutting edge technologies, such as next generation sequencing, are quickly being adapted into epidemiologic investigations, particularly for cases and outbreaks of unknown origin, although older, mature technologies with larger existing databases will still be needed for appropriate comparative analyses.

## Introduction

An apparent autochthonous case of melioidosis was identified in rural southeastern AZ, U.S., in October 2008 [1]. The patient, with no prior travel history to endemic regions, was confirmed as being infected with *Burkholderia pseudomallei*, the etiological agent of melioidosis. As no endemic cases of melioidosis have been identified in the United States previous to this case, it is likely that a contaminated commodity was the source of this local infection.

### Background

*B. pseudomallei*, a Gram-negative bacillus, is a soil saprophyte capable of surviving harsh environmental conditions. Melioidosis is a serious disease caused by infection with *B. pseudomallei* via inoculation, aspiration or ingestion, resulting in high mortality rates [2]. It is considered a Biosafety Level 3 organism due to risks of aerosolization and severe disease and it is on the U.S. Federal list of Select Agents [3]. It is found in soils throughout Southeast Asia and northern Australia, with sporadic populations and infections found in many parts of the world, except North America and Europe [4]. *B. pseudomallei* is closely related to a number of other *Burkholderia* species and suspect cases in non-endemic regions need to be genetically confirmed to rule out misidentification of the pathogenic agent. Here we describe a molecular investigation of the first reported [1] instance of apparent non-human-to-human autochthonous transmission of *B. pseudomallei* in the United States.

### Case Information

In October of 2008, a 32-year-old diabetic male was admitted to a rural Arizona hospital with severe knee pain and an initial diagnosis of *E. coli* septicemia [1]. The patient did not improve after eight days of antimicrobial therapy and was transferred to a large acute care hospital. Blood cultures at the second hospital grew suspect *B. pseudomallei*, via automated biochemical analysis (Vitek, bioMerieux, France). Subsequent sputum and knee aspirate samples also grew apparent *B. pseudomallei* cultures. These results were confirmed at the Arizona State Public Health Laboratory (Phoenix, AZ), through the use of the Laboratory Response Network real-time PCR protocols for *B. pseudomallei* [5], and subsequently at the U.S. Centers for Disease Control and Prevention (CDC) (Atlanta, GA). As the patient had no travel or other exposure history and due to the lack of endemic transmission of *B. pseudomallei* in the United States, advanced molecular studies were conducted to assist with the investigation.

## Methods

### Samples

Only one original culture isolate from the patient was available for molecular analysis. No additional isolates were identified during the course of the epidemiologic investigation involving patient contacts, pets, home-site soil and imported plants. The case isolate was provided by the Arizona State Health Laboratory and was handled, extracted and stored in the select agent certified BSL3 laboratory at Northern Arizona University (Flagstaff, AZ). The isolate was streaked on Ashdown's agar and incubated at 37°C for 48 hr. DNA was extracted from bacterial growth using Wizard genomic DNA purification kit (Promega, USA), using manufacturer's protocol. Sterility tests were performed on the DNA samples prior to removal from bio-safety containment.

### PCR Profiling

PCR assays targeting the genetic loci listed in Table 1 were performed [6]–[10] (see Table S1 for specific assay primer/probe sequences). These assays include species detection for *B. pseudomallei* (TTS1, *bimA*_Bp_, BurkDiff-Bp) [6], [9], [10]; *B. mallei*, the etiologic agent of glanders and also known as a recent clone of *B. pseudomallei* (*bimA*_Bm_, BurkDiff-Bm) [9], [10]; and *B. thailandensis*, a common soil species that rarely causes human infection (*cheB*) [7]. Additionally, we targeted known virulence (*wcbG*, *bpaA*, *fhaB*_1-3) and metabolic (BPSS0654) genes [8]. Lastly, we also included real-time PCR assays to a pair of genetic targets that distinguishes the Southeast Asian *B. pseudomallei* (YLF) from Australian (BTFC) populations [7]. Real-time PCR was carried out in a 384-well plate in 10 uL reactions containing 900 nM of forward and reverse primers, 250 nM of probe for single-assay reactions or 200 nM of each probe for duplex reactions, 1× Applied Biosystems Universal Mastermix (Life Technologies, Carlsbad, CA), and 0.5 ng template. 1× Applied Biosystems Genotyping Mastermix (Life Technologies) was used in place of Universal Mastermix for the BurkDiff assay [10]. Thermal cycling and endpoint analysis was performed on an AB 7900HT sequence detection system (Life Technologies) using the following conditions: 50°C for 2 min, 95°C for 10 min, and 40 cycles of 95°C for 15 s and 58°C for 1 min. Positive controls for each assay are listed in Table S2 and negative controls consisted of several near neighbor (i.e., strains of non-*B. psuedomallei Burkholderia* species) and non-related bacterial strains along with “no template” controls.

**Table 1:** Real-time PCR assay panel and results used to profile case isolate.

Genomic target	Result	Specificity	Target info
TTS1	Pos.	*B.ps.*	Type III secretion gene cluster [6]
YLF	Pos.	SEA isolates of *B.ps.*	Yersinia-like fimbrial gene cluster; mutually exclusive of BTFC; mostly found in *B.ps.* from Thailand [7]
BTFC	Neg.	Australian isolates of *B.ps.*	*B. thailandensis*-like flagella & chemotaxis gene cluster; mutually exclusive of YLF; mostly found in *B. ps.* from Australia [7]
*cheB*	Neg.	*B. thailandensis*	*B. thailandensis* homolog of BTFC [7]
*wcbG*	Pos.	*B.ps.* virulence gene	A gene in the *wcb* capsule biosynthesis operon; more prevalent in clinical isolates than environmental isolates of *B. ps.* (A. Tuanyok, unpublished data)
*fhaB*_1	Pos.	*B.ps.* virulence gene	Filamentous hemagglutinin (adhesin; a virulence molecule produced by intracellular bacteria). B.ps. *fhaB* genes vary in sequence and copy number among strains and may infer geographic origin. [8]
*fhaB*_2	Pos.	*B.ps.* virulence gene	
*fhaB*_3	Pos.	*B.ps.* virulence gene	
*bimA*_Bm_	Neg.	Most *B.m.*, some *B.ps.*	*Burkholderia* intracellular motility A protein (a virulence factor) – *B. mallei* dominant [9]
*bimA*_Bp_	Neg.	Most *B.ps.*	*Burkholderia* intracellular motility A protein (a virulence factor) – *B. pseudomallei* dominant [9]
*bpaA*	Neg.	*B.ps.* virulence gene	Virulence gene located on a genomic island [8]
BPSS0654	Pos.	*B.ps.* metabolic gene	Common metabolic gene on genomic islands [8]
BurkDiff	*B.ps.* Pos.*B.m.* Neg.	*B.m.* and *B.ps.*	Single nucleotide polymorphism differentiating *B.m.* from *B.ps.* Flanking region is unique to these two species. (10)

(*B.ps.* = *B. pseudomallei*; *B.m.* = *B. mallei*; Pos. = Positive; Neg. = Negative; SEA = Southeast Asia).

### MLST Genotyping

We conducted multi-locus sequence typing (MLST) for standardized genotyping, as previously described [11]. The identified allele profile was assigned a sequence type (ST) based on the naming conventions described in the MLST database [12]. We then used *Structure* [13] to conduct population genetic analyses comparing identified sequence types to the MLST database, to better understand the likelihood of broad geographic origination.

### Whole Genome Sequencing

To further detail the genomic features of the case strain and to compare to *B. pseudomallei* genome sequences, we conducted whole genome sequence analysis, using next generation sequence technology. DNA libraries were prepared for sequencing on the Illumina Genome Analyzer II, using a protocol provided by Illumina (San Diego, CA, USA). About 2 µg of DNA was sheared with the SonicMAN™ (Part # SCM1000-3, Matrical Bioscience, Spokane, WA) to an average size of 600 base pairs with the following parameters: Sonication - 10 sec; Power -100%. The remainder of the preparation followed the Illumina protocol (“Preparing Samples for Multiplexed Paired-End Sequencing”, Catalog #PE-930-1002, Part #1005361). The library was quantified using the Bioanalyzer per the Illumina protocol. The paired end library was sequenced on the Illumina GAII platform to a length of 50 base pairs per read.

The sequenced reads were aligned to *Burkholderia pseudomallei* K96243 chromosomes 1 and 2 (Genbank ID's BX571965 and BX571966, respectively) using Burrows-Wheeler Aligner [14]. Insertions and deletions were removed as well as alignments that mapped to multiple locations. SolSNP (http://sourceforge.net/projects/solsnp/) was used to identify SNPs from the alignment file. SNPs that were not present in 90% of the base calls at each position and did not meet a minimum coverage of 10× were removed. Publically available genomes were aligned against K96243 using MUMmer 3.22 [15]. SNPs were identified from the alignments using a custom script, allowing for placement in a well-established phylogeny [16]. SNPs within duplicated regions were identified with MUMmer 3.22 and removed from the phylogenetic analysis. Alleles lacking coverage were removed as well. The maximum parsimony algorithm in MEGA5 [17], with the average pathway method to determine branch lengths [18], was used for phylogenetic analysis. Where shown, bootstrap analysis was performed with 1000 generations.

## Results

### PCR-Profiling

As listed in Table 1, the detection assays (TTS1, *bimA*_Bp_, BurkDiff-Bp) were positive for *B. pseudomallei*, and the detection assays for *B. mallei* (*bimA*_Bm_, BurkDiff-Bm) and *B. thailandensis* (*cheB*) were negative. The YLF+ versus BTFC- results grouped the patient isolate with Southeast Asia populations. The profile of virulence gene assay results (*fhaB*_1+, *B*_2+ and *B*_3 +, *wcbG*+, *bpaA*−) is rare, seen only previously in limited environmental isolates and clinical samples in Southeast Asia [8].

### MLST

**Figure 1:**
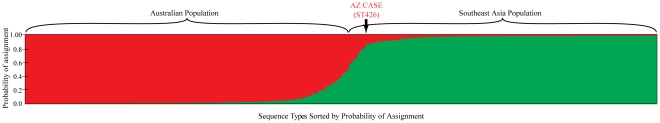
Population assignment of case isolate based on MLST sequence type. An estimated population assignment of case isolate (ST426) compared to 640 other MLST sequence types was developed using *Structure* [13]. Each thin vertical line represents one sequence type (ST) and is divided into 2 portions that represent the likelihood of assignment into each population. STs are sorted by likelihood of assignment into two populations. STs from strains collected in Australia dominate the left hand population and STs from strains collected in Southeast Asia dominate the right hand population. The case isolate was placed in the second population in 85% of 100,000 iterations.

When compared to the global MLST database [12], the case isolate alleles were identical to a single previously typed isolate from a patient presenting with severe melioidosis in Australia (ST426). Further epidemiological follow-up determined that the Australian patient was infected in Malaysia. Population genetic analyses placed this isolate with 85% likelihood in the Southeast Asian population (Figure 1).

### Whole Genome Sequencing and Typing (WGST)

**Figure 2:**
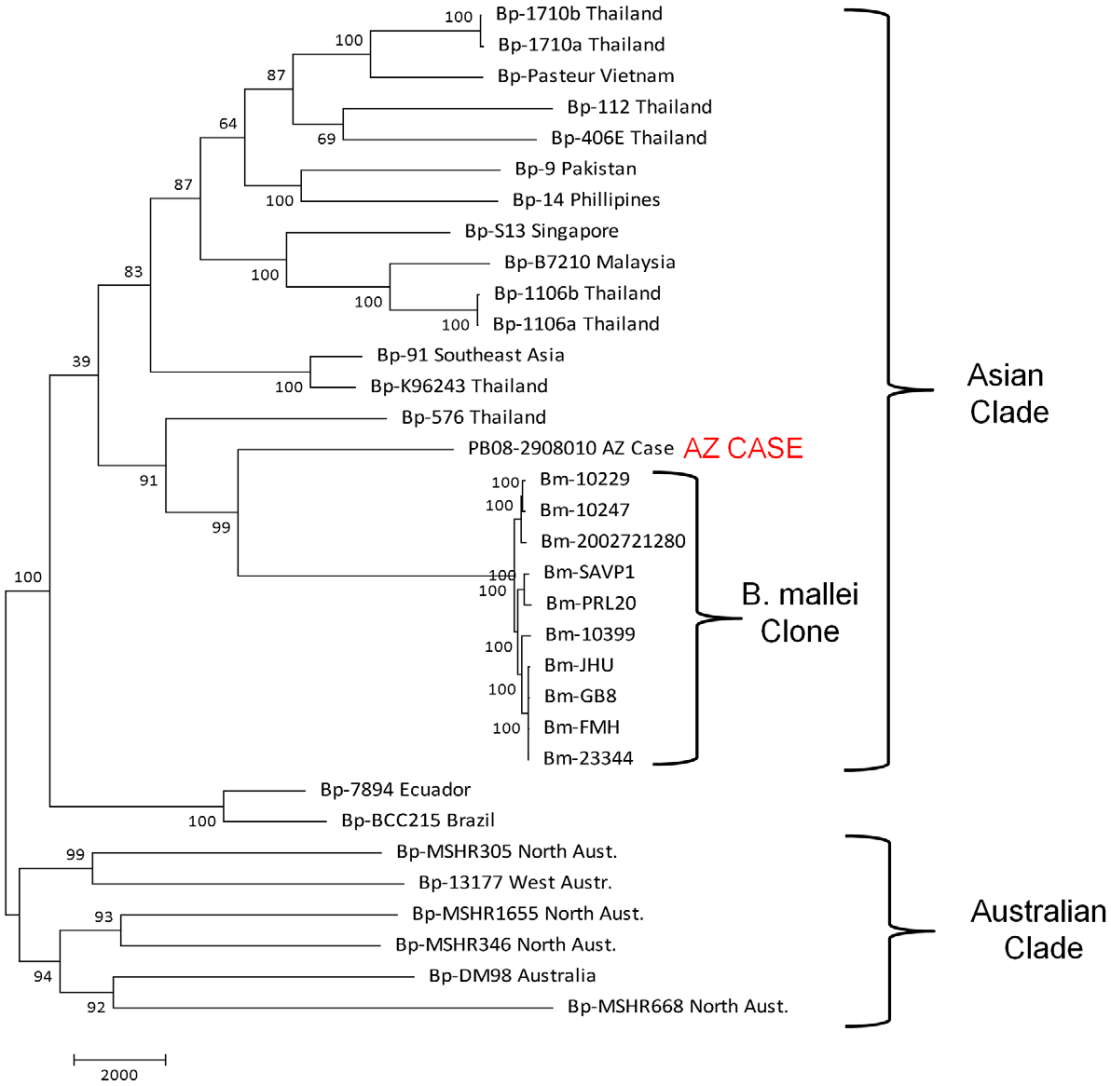
Maximum parsimony phylogenetic comparison of case isolate sequence to all available *B. pseudomallei* sequences. The case isolate whole genome sequence (WGS) was compared to 22 *B. pseudomallei* and 10 *B. mallei* sequences, using 54,135 shared SNPs (including 31,202 parsimony informative SNPs). MEGA5 [17] was used to conduct maximum parsimony analysis of all SNP loci common to the genome sequences. The percentages of replicate trees in which the associated taxa clustered together in the bootstrap test (1000 replicates) are shown next to the branches. The tree is drawn to scale, with branch lengths calculated using the average pathway method [18] and are in the units of the number of changes over the whole sequence.

The sequence run generated 29,517,572 50-base pair paired-end reads (1,476 Mb). The average read length was 44 base pairs. Reads mapped to 88.6% of the K92643 with a minimum of 10× coverage at each base included in the analysis. Using *B. pseudomallei* K96243 strain as the reference, we identified 54,135 orthologous SNP loci, including 31,202 parsimony informative SNPs. When comparing the shared SNP states of the case isolate to the publically available 32 *B. pseudomallei* and *B. mallei* whole genome sequences, a maximum parsimony phylogenetic analysis grouped the case genome with Southeast Asian strains (Figure 2). Interestingly, the case isolate along with a Thai strain (Bp576) are on the branch that contains the *B. mallei* clone. The *B. mallei* clone was previously shown to belong to the Southeast Asian clade [16].

## Discussion

This report describes molecular investigations into the first case of non-human-to-human locally acquired melioidosis infection in the United States; a possible sexually transmitted U.S. autochthonous case associated with a returning Viet Nam veteran in 1971 has been previously described [19]. In this case, without a clear mechanism of transmission, an in-depth molecular analysis was necessary to help understand the possible nature of the exposure and the isolate. Here we used standard and novel tools to confirm the identification of *B. pseudomallei* and further characterize the isolate as most likely originating from materials coming from Southeast Asia, possibly in or around Malaysia. Despite on-site epidemiological investigations [1], no physical or historical evidence was discovered to link the patient to this region of the world.

As *B. pseudomallei* is classified as a Select Agent [3], identification of even an individual case of melioidosis in non-endemic regions requires initial law enforcement notification and rapid epidemiological and forensic analysis to rule out suspect terrorism. The real-time PCR assays used in this investigation provided public health and safety officials with initial information regarding potential source location of the etiological agent – the presence of all three *fhaB* virulence genes and the positive YLF result are typically only seen with Southeast Asian isolates [8]. Ensuing genotyping methodologies confirmed this finding. These initial data also were useful in focusing the on-site epidemiological investigation for possible sources of fomites and/or products associated with the exposure.

As a comparative or source isolate was not identified during the investigation, appropriate genotyping tools were limited. Additionally, given the highly recombinagenic nature of *B. pseudomallei* [20], the connection between genotypes (isolates linked by apparent genetic similarities) and phylotypes (isolates linked by evolutionary relationships) is limited at best. Multiple genotyping systems for *B. pseudomallei*, however, have been published, including pulsed-field gel electrophoresis, randomly amplified polymorphic DNA [21], multi-locus VNTR analysis (MLVA) [22], PCR-based genotyping [7], multi-locus sequence typing (MLST) [12], and more recently, orthologous SNP analysis (WGST) [16]. We employed the latter three systems, or variations thereof, for this investigation to achieve the highest molecular epidemiologic confidence possible for this unusual melioidosis case. These analyses have different species and strain resolution powers and varying support from genetic databases. The greatest resolution can be achieved through WGST (using thousands of loci), but, due to the just-recent application of this technology to molecular epidemiology, this system has limited comparative strain databases. Other methodologies have more limited resolving capacity (*e.g.*, MLST), but are complemented with extensive strain databases. MLVA, although known to have high resolving power, was not used in this investigation, as it is appropriate for comparison of only known closely related isolates [23].

Assigning isolates to specific MLST sequence types allows for the inference of epidemiological linkage between identical genotypes, but has limited use in determining relationships among sequence types [16], restricting its usefulness for phylogeographic analysis, and in turn, molecular epidemiology. Nevertheless, MLST has proved very discriminatory when comparing large *B. pseudomallei* isolate sets from across melioidosis-endemic northern Australia, with no ST yet found to be common to both the Northern Territory and Queensland [24]. Furthermore, when correct attribution of location of origin of *B. pseudomallei* strains is confirmed, no MLST ST has to date been found to be common to both Australia and Southeast Asia [25], allowing for inference of larger scale geographic origin based on sequence type [16]. The exact MLST match of the case with only one other isolate in the global MLST database (ST426 – isolate #MSHR158 from case exposed in Malaysia) and the MLST population genetic analysis provides substantial evidence of the originating location.

Due to the limitations of MLST in determining relationships among sequence types, we were restricted in our ability to further refine our estimates of geographic origin using this technique. We therefore explored more loci-intensive genotyping systems, microarray SNP screening and WGST, using SNPs derived from whole genome analyses. SNP-based genotyping is known to be highly accurate in terms of defining population subgroups and isolate relationships, especially in clonal organisms [26]. SNPs are considered to be more phylogenetically informative than other molecular markers (*e.g.*, VNTRs) due to their typically biallelic nature, slow mutation rates and genome-wide distribution [27]. However, the use of SNPs for phylogenetic analysis in *B. pseudomallei* is not straightforward due to character state conflicts arising from genetic recombination via lateral gene transfer. No significant consensus was identified from phylogenetic analysis of a moderate number of orthologous SNP loci (n<500), screened via microarray, due to this described homoplasy (See Text S1 and Figure S1). However, the use of large SNP data sets has been shown to mitigate this challenge [16], [28]. WGST was used to compare the case isolate genome to all existing whole genome sequences. The strength of this analysis is in the comparison of all potential differences and similarities in the genome (in this case, nearly 30,000 orthologous SNPs), thereby establishing evolutionary lineages and providing the best evidence of phylogenetic relationships. The limitation of this analysis is the relatively small number of whole genomes available for comparative analysis (n = 23). This limited number of whole genomes prevents more fine-scale phylogeographic inference, primarily due to likely branch collapse caused by selection bias [26]. In other words, a bias is introduced by the selection or availability of strains that may not represent the true phylogenetic diversity of an organism. The result is collapsed secondary branches in the structure that lead to strains that have not been sequenced yet [26]. As with the other genotyping tools used in this study, WGST phylogenetic analysis placed the clinical isolate among isolates from Southeast Asia. The association of the strain with the pre-*B. mallei* branch requires further analysis, however this finding is not surprising given previous discovery that the *B. mallei* species is a clone found in the Southeast Asian *B. pseudomallei* population [16].

It is possible that this patient had a low dose exposure to *B. pseudomallei*, as has been described previously [29], [30], limiting the success of the subsequent epidemiological investigations in finding the source, whether in the environment or contaminated products. Future epidemiological studies may have better success in identifying environmental sources with continued improvement of technologies for sensitive detection of limited or remnant DNA.

Next generation and third generation sequencing technologies can be expected to be more widely used in epidemiological investigations as costs decrease and speed, coverage and accuracy increase [28], [31]–[33]. However, other molecular methods are needed to bridge the gap between vast amounts of data gathered from a small number of strains and fewer data from a large number of isolates. Indeed, a comparison of clinical strains to a large database of molecular data from a diverse and comprehensive strain collection is needed for precise attribution. For *B. pseudomallei*, recombination among strains complicates attribution efforts requiring large numbers of loci for accurate determination of strain relatedness. Our use of the large *B. pseudomallei* MLST database, PCR profiling, and WGST all provide evidence with complementary strengths and weaknesses that point to a Southeast Asian ancestry for the clinical isolate. Future investigations can and should use next generation sequencing data for both WGST and MLST (which can be extracted from the assembled/aligned sequence reads), as the cost of reagents is now around $100 (US) per isolate (for similar analyses described here). Additionally, the availability of whole genome data will allow for additional comparative genomics studies that may provide additional clinical, public health, biodefense and basic science benefit. As next generation instruments and bioinformatic tools continue to become more user-focused and routine, sequence databases are becoming more robust and use of whole genome data will feasibly, and necessarily, become a standard for molecular epidemiology. This will provide improved ability to quickly link isolates together, understand phylogeographic relationships, and determine outbreak source.

## Supporting Information

Figure S1 ***B. pseudomallei*** **microarray SNP phylogeny.** A phylogeny (neighbor joining tree) derived from 151 SNPs from 108 available *B. pseudomallei*, *B. mallei*, and *B. thailandensis* whole genomes was derived from the SNP microarray analysis. The tree is drawn to scale with branch lengths calculated using the average pathway method and are in the units of the number of changes over the whole sequence. Due to high levels of recombination and subsequent homoplasy (SNPs accruing in evolutionarily independent groupings) present, no statistical calculations were performed. Analysis includes approximately 20 replicates for precision control. Map generated from *maps.google.com*.
(JPG)

Table S1 **Primer and probe design and amplicon size for PCR assays.**
(DOC)

Table S2 **Positive control strains by assay.**
(DOC)

Text S1 **Supplemental methods and results for SNP microarray analysis.**
(DOC)

## References

[1] Stewart T, Engelthaler DM, Blaney D, Tuanyok A, Wangsness E (2011). Epidemiology and Investigation of Melioidosis in Southern Arizona.. Emerg Infect Dis.

[2] Peacock SJ (2006). Melioidosis.. Curr Opin Infect Dis.

[3] Rotz LD, Khan AS, Lillibridge SR, Ostroff SM, Hughes JM (2002). Public health assessment of potential biological terrorism agents.. Emerg Infect Dis.

[4] Cheng AC, Currie BJ (2005). Melioidosis: epidemiology, pathophysiology, and management.. Clin Microbiol Rev.

[5] Centers for Disease Control and Prevention. 11 March 2005, posting date. Laboratory Preparedness for Emergencies.. http://www.bt.cdc.gov/lrn/.

[6] Novak RT, Glass MB, Gee JE, Gal D, Mayo MJ (2006). Development and Evaluation of a Real-Time PCR Assay Targeting the Type III Secretion System of Burkholderia pseudomallei.. J Clin Microbiol.

[7] Tuanyok A, Auerbach RK, Brettin TS, Bruce DC, Munk AC (2007). A horizontal gene transfer event defines two distinct groups within Burkholderia pseudomallei that have dissimilar geographic distributions.. J Bacteriol.

[8] Tuanyok A, Leadem BR, Auerbach RK, Beckstrom-Sternberg SM, Beckstrom-Sternberg JS (2008). Genomic islands from five strains of Burkholderia pseudomallei.. BMC Genomics.

[9] Ulrich MP, Norwood DA, Christensen DR, Ulrich RL (2006). Using real-time PCR to specifically detect Burkholderia mallei.. J Med Microbiol.

[10] Bowers JR, Engelthaler DM, Ginther JL, Pearson T, Peacock SJ (2010). BurkDiff: A Real-Time PCR Allelic Discrimination Assay for Burkholderia pseudomallei and B. mallei.. PLoS One.

[11] Godoy D, Randle G, Simpson AJ, Aanensen DM, Pitt TL (2003). Multilocus sequence typing and evolutionary relationships among the causative agents of melioidosis and glanders, Burkholderia pseudomallei and Burkholderia mallei.. J Clin Microbiol.

[12] Multi Locus Sequence Typing – Burkholderia pseudomallei MLST [Internet].

[13] Pritchard JK, Stephens M, Donnelly P (2000). Inference of population structure using multilocus genotype data.. Genetics.

[14] Li H, Durbin R (2009). Fast and accurate short read alignment with Burrows-Wheeler Transform.. Bioinformatics.

[15] Kurtz S, Phillippy A, Delcher AL, Smoot M, Shumway M (2004). Versatile and open software for comparing large genomes.. Genome Biology.

[16] Pearson T, Giffard P, Beckstrom-Sternberg S, Auerbach R, Hornstra H (2009). Phylogeographic reconstruction of a bacterial species with high levels of lateral gene transfer.. BMC Biol.

[17] Tamura K, Peterson D, Peterson N, Stecher G, Nei M (2011). MEGA5: Molecular Evolutionary Genetics Analysis using Maximum Likelihood, Evolutionary Distance, and Maximum Parsimony Methods. Molecular Biology and Evolution.. Mol Biol Evol.

[18] Nei M, Kumar S (2000). Molecular Evolution and Phylogenetics.

[19] McCormick JB, Sexton DJ, McMurray JG, Carey E, Hayes P (1975). Human-to-human transmission of Pseudomonas pseudomallei.. Ann Intern Med.

[20] Holden MTG, Titball RW, Peacock SJ, Cerdeno-Tarraga AM, Crossman LC (2004). Genomic plasticity of the causative agent of melioidosis, Burkholderia pseudomallei.. Proc Natl Acad Sci USA.

[21] Ulett GC, Currie BJ, Clair TW, Mayo M, Ketheesan N (2001). Burkholderia pseudomallei virulence: definition, stability and association with clonality.. Microbes Infect.

[22] U'Ren JM, Schupp JM, Pearson T, Hornstra H, Friedman CL (2007). Tandem repeat regions within the Burkholderia pseudomallei genome and their application for high resolution genotyping.. BMC Microbiol.

[23] Pearson T, U'Ren JM, Schupp JM, Allan GJ, Foster PG (2007). VNTR analysis of selected outbreaks of Burkholderia pseudomallei in Australia.. Infect Genet Evol.

[24] Cheng AC, Ward L, Godoy D, Norton R, Mayo M (2008). Genetic diversity of Burkholderia pseudomallei isolates in Australia.. J Clin Microbiol.

[25] Currie BJ, Thomas AD, Godoy D, Dance DA, Cheng AC (2007). Australian and Thai isolates of Burkholderia pseudomallei are distinct by multilocus sequence typing: revision of a case of mistaken identity.. J Clin Microbiol.

[26] Pearson T, Okinaka RT, Foster JT, Keim P (2009). Phylogenetic understanding of clonal populations in an era of whole genome sequencing.. Infect Genet Evol.

[27] Keim P, Van Ert MN, Pearson T, Vogler AJ, Huynh LY (2004). Anthrax molecular epidemiology and forensics: using the appropriate marker for different evolutionary scales.. Infect Genet Evol.

[28] Engelthaler DM, Chiller T, Schupp JA, Colvin J, Beckstrom-Sternberg SM (2011). Next-generation sequencing of Coccidioides immitis isolated during cluster investigation.. Emerg Infect Dis.

[29] Jeddeloh JA, Fritz DL, Waag DM, Hartings JM, Andrews GP (2003). Biodefense-driven murine model of pneumonic melioidosis.. Infect Immun.

[30] Ulett GC, Labrooy JT, Currie BJ, Barnes JL, Ketheesan N (2005). A model of immunity to Burkholderia pseudomallei: unique responses following immunization and acute lethal infection.. Microbes Infect.

[31] Cummings CA, Bormann Chung CA, Fang R, Barker M, Brzoska PM (2009). Whole-genome typing of Bacillus anthracis isolates by next-generation sequencing accurately and rapidly identifies strain-specific diagnostic polymorphisms.. Forensic Sci Int Genet.

[32] Harris SR, Feil EJ, Holden MT, Quail MA, Nickerson EK (2010). Evolution of MRSA during hospital transmission and intercontinental spread.. Science.

[33] Lienau EK, Strain E, Wang C, Zheng J, Ottesen AR (2011). Identification of a Salmonellosis Outbreak by Means of Molecular Sequencing.. N Engl J Med.

