# Supplementary figures and images for: Molecular Investigations of a Locally Acquired Case of Melioidosis in Southern AZ, USA
Source: PLoS Negl Trop Dis. 2011 Oct 18;5(10):e1347. doi: 10.1371/journal.pntd.0001347 (PMC3196475; doi:10.1371/journal.pntd.0001347)

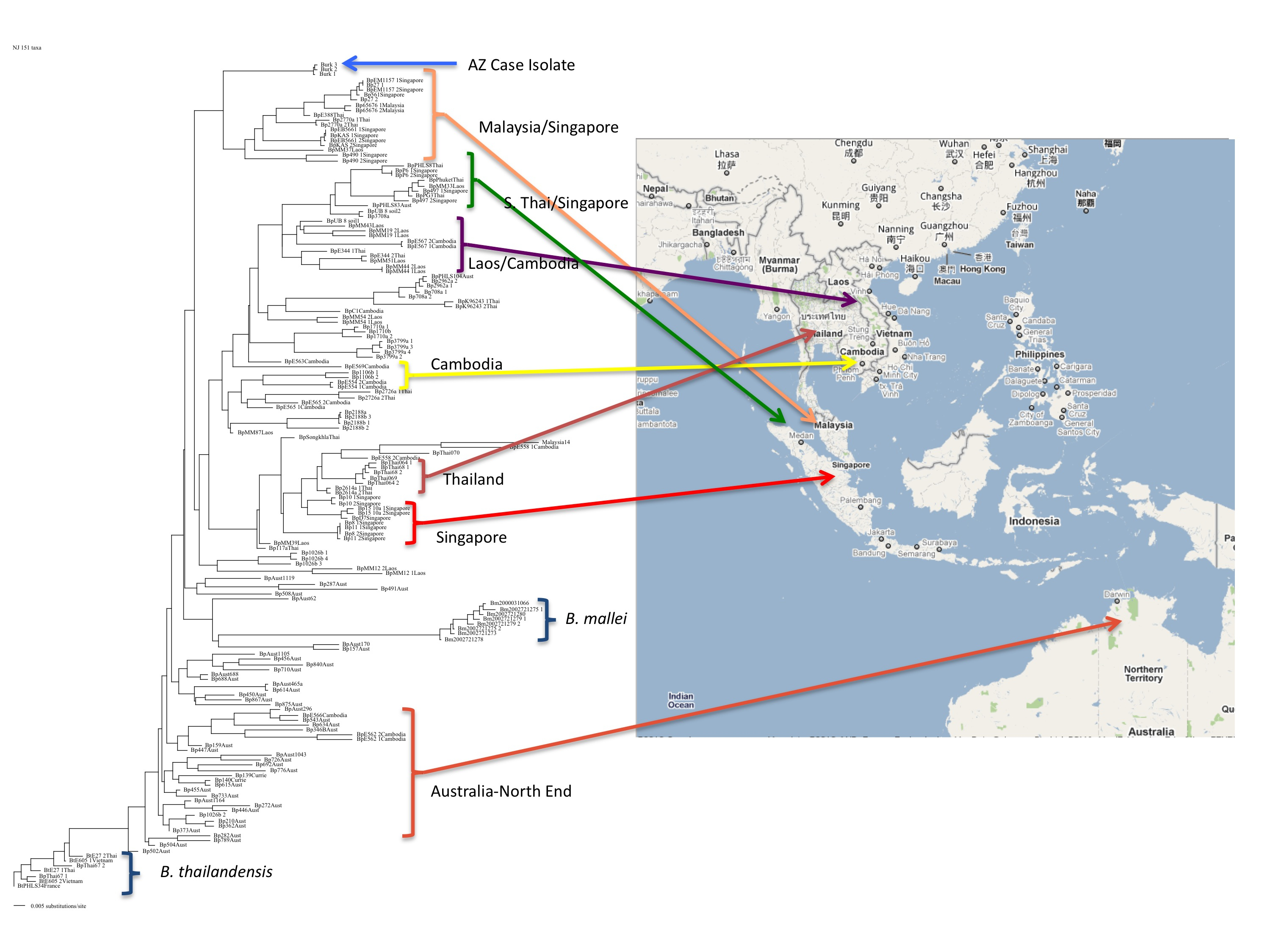

Supplement: Figure S1 — B. pseudomallei microarray SNP phylogeny. A phylogeny (neighbor joining tree) derived from 151 SNPs from 108 available B. pseudomallei, B. mallei, and B. thailandensis whole genomes was derived from the SNP microarray analysis. The tree is drawn to scale with branch lengths calculated using the average pathway method and are in the units of the number of changes over the whole sequence. Due to high levels of recombination and subsequent homoplasy (SNPs accruing in evolutionarily independent groupings) present, no statistical calculations were performed. Analysis includes approximately 20 replicates for precision control. Map generated from maps.google.com. (JPG) [file pntd.0001347.s001.jpg]
