# Supplementary material for: Molecular Investigations of a Locally Acquired Case of Melioidosis in Southern AZ, USA
Source: PLoS Negl Trop Dis. 2011 Oct 18;5(10):e1347. doi: 10.1371/journal.pntd.0001347 (PMC3196475; doi:10.1371/journal.pntd.0001347)
Supplement: Table S1 — Primer and probe design and amplicon size for PCR assays. (DOC) [file pntd.0001347.s002.doc]

Table S1. Primer and probe design and amplicon size for PCR assays.

| Assay Name | Primer/Probe Name | Sequence | Amplicon size (bp) |
| --- | --- | --- | --- |
| TTS1 | BpTT4176F | CGTCTCTATACTGTCGAGCAATCG | 115 |
|  | BpTT4290R | CGTGCACACCGGTCAGTATC |
|  | BpTT4208P | FAM-CCGGAATCTGGATCACCACCACTTTCC-BHQ |
| YLF | YLF_F | CCGGGCCTTTCATGCTGTC | 73 |
|  | YLF_R | TGTTCGGTGATTTCGATTTGGA |
|  | YLF_p | FAM-ATCGGCAGCGTCGCGCAGAA-BHQ |
| BTFC | BTFC_F | CGAGCGCGTGAATCGAGTTG | 95 |
|  | BTFC_R | CGACTGATCGCCAATTTCCA |
|  | BTFC_p | CAL Fluor Gold 540-CATGGTGATTCGTCAAAGCCGTCGC-BHQ |
| cheB | cheB_F | ATCGGCCGGAGACGATTT | 74 |
|  | cheB_R | ACTACGCGAATCAATTCGTTTTC |
|  | cheB_p | FAM-CTCCGCCGTTCTCGACTGCAAAAAC-BHQ |
| wcbG | wcbG_F | ACACGCCCGCTGATTCCAA | 79 |
|  | wcbG_R | GGTCCGGCATCGAGGATT |
|  | wcbG_p | FAM-TAATCTGCTCGCGCACCTGCA-BHQ |
| fhaB genes | fhaB1_F | CGCCTTGGACGGTCACAT | 124 |
|  | fhaB1_R | TCGCTCAATACCGATGGGATG |
|  | fhaB1_p | CAL Fluor Gold 540-CCGTTGCCGCCGATCACACC-BHQ |
|  | fhaB2_F | GCTCCCGTTGGGCTCATAC | 89 |
|  | fhaB2_R | TCAGGCTTCACCCAGATGATG |
|  | fhaB2_p | FAM-CGAGGCTTTGCAAGGCTTGCTC-BHQ |
|  | fhaB3_F | GGCGGCTTCCTCAATACGAG | 97 |
|  | fhaB3_R | GGCCTTGGTTGACGTTGAAAC |
|  | fhaB3_p | FAM-AGCCACGCTGACAACCGGCAAT-BHQ |
| bimA | bimA-Bm_F2 | AGCGCTTCGCGCATCTAC | 104 |
|  | bimA-Bm_R2 | CGCGTTAAACGCCGTACTTTC |
|  | bimA-Bm_BHQ | CAL Fluor Gold 540-TGTTCGTCCATCGCTCCGCTGG-BHQ |
|  | bimA-Bps_F2 | CTCGCTCGCCGGATCAAG | 58 |
|  | bimA-Bps_R2 | GCTTTGGCGTGCATATCGA |
|  | bimA-Bps_BHQ | FAM-CCATGCCTTCCTCGACTAATCCCAC-BHQ |
| bpaA | bpaA_F | TGGACAACAATCAAGGCAATG | 99 |
|  | bpaA_R2 | TGCTCATCGGCACATAGTTGATG |
|  | bpaA_FAMBHQ | FAM-CAGTGCCAACGATCTGACCAATCTGC-BHQ |
| BPSS0654 | bpss0654_F | TGGATGGGCACGACGTTAC | 63 |
|  | bpss0654_R | CGACATCCAGCCCCTTGTAG |
|  | BPSS0654-FAMBHQ | FAM-AAGCGCGGATTTGCCCAGCC-BHQ |
| BurkDiff | BD_F | CGAGCGCATCGTACTCGTA | 73 |
|  | BD_R | CAAGTCGTGGATGCGCATTA |
|  | BD_FAM-Bm-C | FAM-CTGAAACGCGCAGCG-MGBNFQ |
|  | BD_VIC-Bps | VIC-CTGAAACGCGAAGCG-MGBNFQ |
