# Supplementary material for: Molecular Investigations of a Locally Acquired Case of Melioidosis in Southern AZ, USA
Source: PLoS Negl Trop Dis. 2011 Oct 18;5(10):e1347. doi: 10.1371/journal.pntd.0001347 (PMC3196475; doi:10.1371/journal.pntd.0001347)
Supplement: Table S2 — Positive control strains by assay. (DOC) [file pntd.0001347.s003.doc]

| Assay Name | Control Strain | | | | |
| --- | --- | --- | --- | --- | --- |
|  | Strain ID | Species* | Sample Type | Geographic Origin | Year Collected |
| TTS1 | MSHR305 | B.p | Human | Northern Territory, Aus | 1994 |
| BTFC | “ | “ | “ | “ | “ |
| YLF | 346 | B.p. | Human | Northern Territory, Aus | 1995 |
| cheB | E264 | B.t. | Unk | Unk | Unk |
| wcbG | 2002721785 | B.p. | Environment | Thailand | 1965 |
| fhaB1 | “ | “ | “ | “ | “ |
| fhaB2 | “ | “ | “ | “ | “ |
| fhaB3 | “ | “ | “ | “ | “ |
| bimA_Bm | “ | B.m. | Unk | France | 1972 |
| bimA_Bp | MSHR305 | B.p. | Human | Northern Territory, Aus | 1994 |
| bpaA | “ | “ | “ | “ | “ |
| BPSS0654 | “ | “ | “ | “ | “ |
| BurkDiff_Bm | 2002721280 | B.m. | Unk | France | 1972 |
| BurkDiff_Bp | MSHR305 | B.p. | Human | Northern Territory, Aus | 1994 |

Table S2. Positive Control Strains by Assay.

*B.p. = *B. pseudomallei*; B.m. = *B. mallei*; B. t. = *B. thailandensis*
