# Supplementary material for: Molecular Investigations of a Locally Acquired Case of Melioidosis in Southern AZ, USA
Source: PLoS Negl Trop Dis. 2011 Oct 18;5(10):e1347. doi: 10.1371/journal.pntd.0001347 (PMC3196475; doi:10.1371/journal.pntd.0001347)
Supplement: Text S1 — Supplemental methods and results for SNP microarray analysis. (DOC) [file pntd.0001347.s004.doc]

Text S1

Supplemental Methods and Results for SNP Microarray Analysis

A novel molecular inversion probe (MIP) SNP-genotyping microarray (Affymetrix, Santa Clara, CA) (1) was used employing 151 *B. pseudomallei* probes developed using. *B. pseudomallei* SNPs, identified from analysis of available whole genome sequences. A total of one hundred diverse *B. pseudomallei*, five *B. mallei*, and three *B. thailandensis* isolates had been previously screened across these SNPs, allowing for the development of a phylogenetic tree, demonstrating phylogeographic separation. The Arizona case isolate was run in triplicate across the array. Phylogenetic analyses grouped each replicate together on a single branch within a clade of *B. pseudomallei* that originated from Malaysia and Singapore (Figure S1), providing further evidence of the original location of the isolate strain. Although the tree lacks statistical support, it demonstrates evidence of appropriate phylogeographic clustering of genomes including the isolation of *B. mallei* as a distinct clonal group and separation of Australian and SEA isolates, as previously described (2).

Literature Cited – Supplemental Methods

1. Hardenbol P, Banér J, Jain M, Nilsson M, Namsaraev EA, Karlin-Neumann GA, et al. (2003) Multiplexed genotyping with sequence-tagged molecular inversion probes. Nat Biotechnol. 21:673-8.
2. Pearson T, Giffard P, Beckstrom-Sternberg S, Auerbach R, Hornstra H, Tuanyok A, et al. (2009) Phylogeographic reconstruction of a bacterial species with high levels of lateral gene transfer. BMC Biol. 18:78.
